# Elevated kinesin family member 26B is a prognostic biomarker and a potential therapeutic target for colorectal cancer

**DOI:** 10.1186/s13046-015-0129-6

**Published:** 2015-02-05

**Authors:** Jingtao Wang, Feifei Cui, Xiao Wang, Yingming Xue, Jian Chen, Yang Yu, Huijun Lu, Meng Zhang, Huamei Tang, Zhihai Peng

**Affiliations:** Department of General Surgery, First People’s Hospital, Shanghai Jiao Tong Univerisity, 85 Wujin Road, Shanghai, 200080 China; Department of Pathology, First People’s Hospital, Shanghai Jiao Tong Univerisity, 85 Wujin Road, Shanghai, 200080 China

**Keywords:** Colorectal cancer, Kinesin family protein 26B, Prognosis, Proliferation

## Abstract

**Background:**

Kinesins play a key role in the development and progression of many human cancers. The present study investigated the expression and clinical significance of kinesin family member 26B (KIF26B) in colorectal cancer (CRC).

**Methods:**

Using quantitative real-time PCR and Western blot analyses as well as immunohistochemical staining of a tissue microarray we examined KIF26B mRNA and protein levels in CRC tumor tissues and paired adjacent normal mucosa. Moreover, the effect of KIF26B knockdown on CRC cell proliferation was investigated using Cell Counting Kit-8 assays.

**Results:**

Expression of KIF26B was found to be elevated in CRC. Suppression of KIF26B inhibited CRC cell proliferation. Furthermore, upregulated expression of KIF26B was significantly correlated with tumor size (*P* = 0.020), American Joint Committee on Cancer (AJCC) stage (*P* = 0.018), T stage (*P* = 0.026), N stage (*P* = 0.013), and differentiation histology (*P* = 0.047). KIF26B was also shown to be an independent prognostic indicator of overall survival for CRC patients (HR 5.621; 95% CI 2.302–13.730; *P* < 0.001).

**Conclusion:**

Our data indicate that KIF26B plays an important role in colorectal carcinogenesis and functions as a novel prognostic indicator and a potential therapeutic target for CRC.

## Background

Colorectal cancer (CRC) is one of the most devastating malignancies and the third leading cause of cancer mortality worldwide [1,2]. To date, surgical resection remains the major treatment for CRC patients [3]. Many improvements have been made in screening, detection and adjuvant therapy for CRC in recent years [4-6]; however, the long-term survival associated with this malignancy is not satisfactory because of tumor recurrence and metastasis [7,8]. To improve the prognosis of CRC patients, there is a great need to identify efficient new targets for early diagnosis and effective disease management.

The kinesin superfamily proteins (KIFs) are a conserved class of microtubule- or ATP-dependent molecular motor proteins that transport membranous organelles, mRNAs and proteins to specific destinations [9]. To date, 45 kinesins, which are categorized into 14 different families, have been identified in humans [10]. Kinesins are largely classified as C-, N- or M-kinesins, which contain their motor domain at the carboxyl-terminus, at the amino-terminus or in the intervening region, respectively [11]. In general, C- and N-kinesins drive microtubule minus end- and plus end-directed motilities, respectively, while M-kinesins depolymerize microtubules [12]. N-kinesins are in the majority [13]. KIFs are involved in many key cellular functions, including mitosis, meiosis, migration, signal transduction, microtubule polymer dynamics and intracellular transport [14]. Accumulating evidence indicates the importance of KIFs in the regulation of many physiological events, such as brain function, developmental patterning and tumor suppression [15]. In recent years, many studies have suggested that KIFs also play an important role in the development or progression of a number of human cancers [16,17]. Thus, a better understanding of the functions of kinesins may facilitate the identification of biomarkers for early detection, indications of prognosis and even molecular-targeted therapy for cancers.

The KIF26B gene, which is located at 1q44, encodes an N-type kinesin protein. KIF26B along with KIF26A are two members of the kinesin-11 superfamily. KIF26A is an unconventional kinesin and plays an important role in enteric nervous system development by regulating the GDNF-Ret signaling pathway [18], while KIF26B is a downstream target of the zinc finger protein Sall1, and plays an important role in kidney development through regulating the adhesion of mesenchymal cells in contact with ureteric buds [19,20]. In the neuronal system, KIF26B plays a role in the transport of Abelson interactor protein 1 (Abi-1) to different cellular compartments, especially to the postsynaptic density of excitatory synapses [21]. Recently, it has been reported that overexpression of KIF26B is associated with poor prognosis in breast cancer [22]. Increased amplification of KIF26B is also associated with higher progression stage of esophageal adenocarcinoma [23]. Genome-wide copy number analysis revealed that KIF26B is associated with predisposition to colorectal adenoma formation [24]. However, the role of KIF26B in CRC carcinogenesis and progression remains to be elucidated.

To investigate the expression and clinical significance of KIF26B in CRC in the present study, we first evaluated KIF26B expression in CRC specimens and paired adjacent normal mucosa. We then examined the relationship between KIF26B expression, clinicopathological features and patient survival using immunohistochemical analysis of CRC tissue microarrays. By knockdown the expression of KIF26B with small hairpin RNA, we also studied the effects of KIF26B on CRC cell proliferation.

## Materials and methods

### Patients and specimens

Tissue specimens were collected from 40 CRC patients (24 male and 16 female; median age, 63.2 y, range 24–88 y) who had undergone tumor resection without receiving chemotherapy or radiotherapy before surgery at the General Surgery Department of the Shanghai JiaoTong University Affiliated Shanghai First People’s Hospital (China) between November 2013 and March 2014. The diagnoses were confirmed by two pathologists, and the tumor staging was based on pathological findings according to the guidelines of the American Joint Committee on Cancer (AJCC) [25]. All the CRC and paired adjacent normal mucosa specimens were collected in the General Surgery Department under protocols approved by the Institutional Review Boards of Shanghai Jiao Tong University Affiliated Shanghai First People’s Hospital Medical Center. Written informed consent was obtained from all patients. The freshly obtained cancer tissues and adjacent normal mucosa (10 cm distant from the original tumor site) were immediately frozen in liquid nitrogen and stored at -80°C prior to RNA and protein extraction.

### Cell lines and plasmids

The established CRC cell lines LoVo, HT29, Caco2, and RKO were obtained from the Type Culture Collection of the Chinese Academy of Sciences (Shanghai, China). The healthy human colon mucosa cell line, NCM460, was purchased from INCELL (San Antonio, TX, USA). ALL cell lines were maintained at 37°C under a humidified atmosphere containing 5% CO_2_. Cell lines were cultured in DMEM/F12 supplemented with 10% FBS (Gibco, USA). The short hairpin RNA (shRNA) plasmid for KIF26B and the control-shRNA plasmid were purchased from Obio Technology (Shanghai, China). For plasmid transfection, 4 × 10^4^ cells per well in six-plates were cultured overnight and then transfected with plasmids using Lipofectamine 2000 (Invitrogen, CA) as to the manufacturer’s instructions. Stable clones of RKO and HT29 cells expressing KIF26BshRNA or control-shRNA were obtained by puromycin selection. The shRNA target sequence for KIF26B was: 5′-GCAGCAAACACATTCCATACA-3′. The control-shRNA target sequence was: 5′-TTCTCCGAACGTGTCACGT-3′.

### RNA extraction, reverse transcription PCR and quantitative real-time PCR

Total RNA was prepared from cell cultures, fresh primary tumors and normal mucosa of 40 CRC patients using TRIzol reagent (TaKaRa, Japan) according to the manufacturer’s instructions. First strand cDNA was synthesized from 2 μg of total RNA using RevertAid™First Strand cDNA Synthesis Kit (Fermentas, USA). For reverse transcription PCR, the reaction was performed for 30 cycles of 94°C for 1 min, 55°C for 1 min, 72°C for 1 min, and an additional extension at 72°C for 10 min. Then, 10 μl of the PCR product was subjected to a 1% agrose gel electrophoresis with ethidium bromide staining and quantification was performed with Quantity One software (Bio-rad, USA). Quantitative real-time PCR (qRT-PCR) assays were performed using 4 μl of cDNA (1:10 dilution) and SYBR green (TaKaRa) in a total volume of 20 μl using the ABI 7900 Real-time PCR System (ABI, USA). The amplification protocol used was as follows: 95°C for 2 min, and then 95°C for 10 s, 60°C for 30 s, and 72°C for 30 s, with a final extension at 72°C for 30 s. Relative quantities (Δ cycle threshold (Ct) values) were obtained by normalizing to GAPDH. Each reaction was performed in triplicate. The following specific primers were used in the assays: GAPDH, sense 5′-GGAGCGAGATCCCTCCAAAAT-3′ and antisense 5′-GGCTGTTGTCATACTTCTCATGG-3′; KIF26B, sense 5′-GCTGGGAATAAAGAGAGGCTTG-3′ and antisense 5′-ACTCCTCGTATGCTTTCCGGT-3′; Ki67, sense 5′-TTCGCAAGCGCATAACCCA-3′ and antisense 5′- AACCGTGTCACAGTGCCAAA-3′; Cyclin D1, sense 5′-GCTGCGAAGTGGAAACCATC-3′ and antisense 5′-CCTCCTTCTGCACACATTTGAA-3′.

### Western blot assays

Total protein was isolated from tissue samples or cultured cell lines using RIPA lysis buffer (Beyotime Biotechnology, China) and the protein concentration was measured using the BCA protein assay kit (Beyotime Biotechnology). Equal amounts of protein (30 μg) were subjected to 10% sodium dodecyl sulfate-polyacrylamide gel electrophoresis and then transferred onto PVDF membranes. Membranes were blocked in 5% fat-free milk solution containing 0.1% Tween-20 for 1 h at room temperature. Membranes were then incubated with primary detection antibody (1:500 dilution for KIF26B, Abcam, USA; 1:1,000 dilution for GAPDH, Abgent, USA) at 4°C overnight followed by incubation with the secondary detection antibody (1:5,000, Abgent) against rabbit IgG-HRP for 2 h at room temperature. Proteins were detected using ECL reagent (Pierce Biotechnology, USA). Quantification based on grayscale analysis was performed with Quantity One software (Bio-rad, USA).

### Immunohistochemistry on tissue microarrays

The tissue microarray (TMA) containing 88 paired CRC specimens was purchased from Xin Chao Company (Shanghai, China). Tumors were resected between July 2006 and May 2007. The final follow-up was on August 2012, with a median patient follow-up time for survivors of 46.62 months (range, 3–73 months). The samples were obtained from 46 men and 42 women with a mean age of 68.72 years (range, 24–90 years). Tumor staging was carried out according to the AJCC staging criteria [25]. Detailed patient demographic information is shown in Table 1. Sections were dewaxed in xylene and rehydrated in a graded series of ethanols, followed by antigen retrieval with 0.01 M sodium citrate buffer (pH 6.0). Immunohistochemical staining was performed using the primary antibody against KIF26B (1:200; Abcam, USA) or Ki67 (1:500; Epitomics, USA), followed by incubation with the HRP-conjugated secondary detection antibody (DakoCytomation, Glostrup, Denmark). Two specialists who were blinded to patient outcome evaluated the staining independently. For KIF26B, staining intensity was scored as 0 (negative), 1 (weak), 2 (moderate), and 3 (strong). Staining extent was scored as 0 (0%), 1 (1%–25%), 2 (26%–50%), 3 (51%–75%), and 4 (76%–100%) according to the percentage of positively stained cells. The final staining score was calculated by multiplying the staining intensity score by the staining extent score. According to the final score, the specimens were divided into two groups: low (0–6), high (7–12). For Ki67, the percentage of tumor cells that showed nuclear staining was assessed. For analysis, a cut-off was set as the median based on a previous study (24), and the samples were into low (<46% positive tumor cells) or high (≥46%) groups.

**Table 1:** **Relationship between clinicaopathologic parameters and KIF26B or Ki67 protein expression (n = 88)**

**Parameters**	**Total**	**KIF26B**		***P***	**KI67**		***P***
		**Low**	**High**		**Low**	**High**	
Age							
<65	31	11	20		13	18	
≥65	57	24	33	0.544^a^	30	27	0.338^a^
Gender							
Male	46	17	29		23	23	
Female	42	18	24	0.572^a^	20	22	0.823^a^
Location							
Right	37	12	25		17	20	
Others	51	23	28	0.231^a^	26	25	0.641^a^
Tumor size (cm)							
<5	47	24	23		21	26	
≥5	41	11	30	0.020^a^	22	19	0.401^a^
AJCC stage							
I + II	52	26	26		31	21	
III + IV	36	9	27	0.018^a^	12	24	0.015^a^
T stage							
T1 + T2	9	7	2		8	1	
T3 + T4	79	28	51	0.026^b^	35	44	0.014^b^
N stage							
N0	54	27	27		32	22	
N1 + N2	34	8	26	0.013^a^	11	23	0.014^a^
M stage							
M0	86	34	52		42	44	
M1	2	1	1	1.000^b^	1	1	1.000^b^
Differentiation							
Well + Moderate	78	34	44		38	40	
Poor	10	1	9	0.047^b^	5	5	0.939^a^
Vascular invasion							
No	84	34	50		42	42	
Yes	4	1	3	1.000^b^	1	3	0.617^b^

^a^Chi-square test.

^b^Fisher’s exact test.

### CCK-8 assays

Cell Counting Kit-8 (CCK-8) assays were used to evaluate cell proliferation according to the manufacturer’s instructions. Briefly, cells were seeded in 96-well plates (2 × 10^3^ cells/well) in triplicate. At the appropriate time (12, 24, 36, 48, 60, 72, 96 h), the cells were incubated with 10 μl CCK-8 solution for 2 h at 37°C. Absorbance was measured at a wavelength of 450 nm on a Gen5 microplate reader (BioTek, USA).

### Plate colony formation assays

For plate colony formation assays, 800 log-phase cells were seeded in six-well plates and cultured at 37°C under a humidified atmosphere containing 5% CO_2_ for 2 weeks. The cells were then fixed with methyl alcohol for 15 min and stained with Giemsa solution for 20 min. Colonies were then counted and photographed All assays were independently performed in triplicate.

### Statistical analysis

SPSS version19.0 for Windows (SPSS, Chicago, IL, USA) was used for all data analysis. For continues variables, data are expressed as mean ± SD and compared using Student’s *t*-test. The association between KIF26B and Ki67 expression and clinicopathological features was analyzed using the Pearson’s *χ*^2^ or Fisher’s exact tests, when appropriate. The correlation between KIF26B and Ki67 protein expression was analyzed using Spearman’s correlation coefficient test. The Kaplan-Meier method was used to calculate the overall survival (OS) curves. Cox proportional hazards models were used in univariate and multivariate analyses to explore the effects of KIF26B expression and CRC clinicopathological variables on survival. Variables that showed a significant prognostic value in univariate analysis were included in the multivariate analysis model using a backward elimination method. For all tests, a *P*-value < 0.05 was considered to be statistically significant.

## Results

### KIF26B expression levels are significantly upregulated in human colorectal cancer

**Figure 1:**
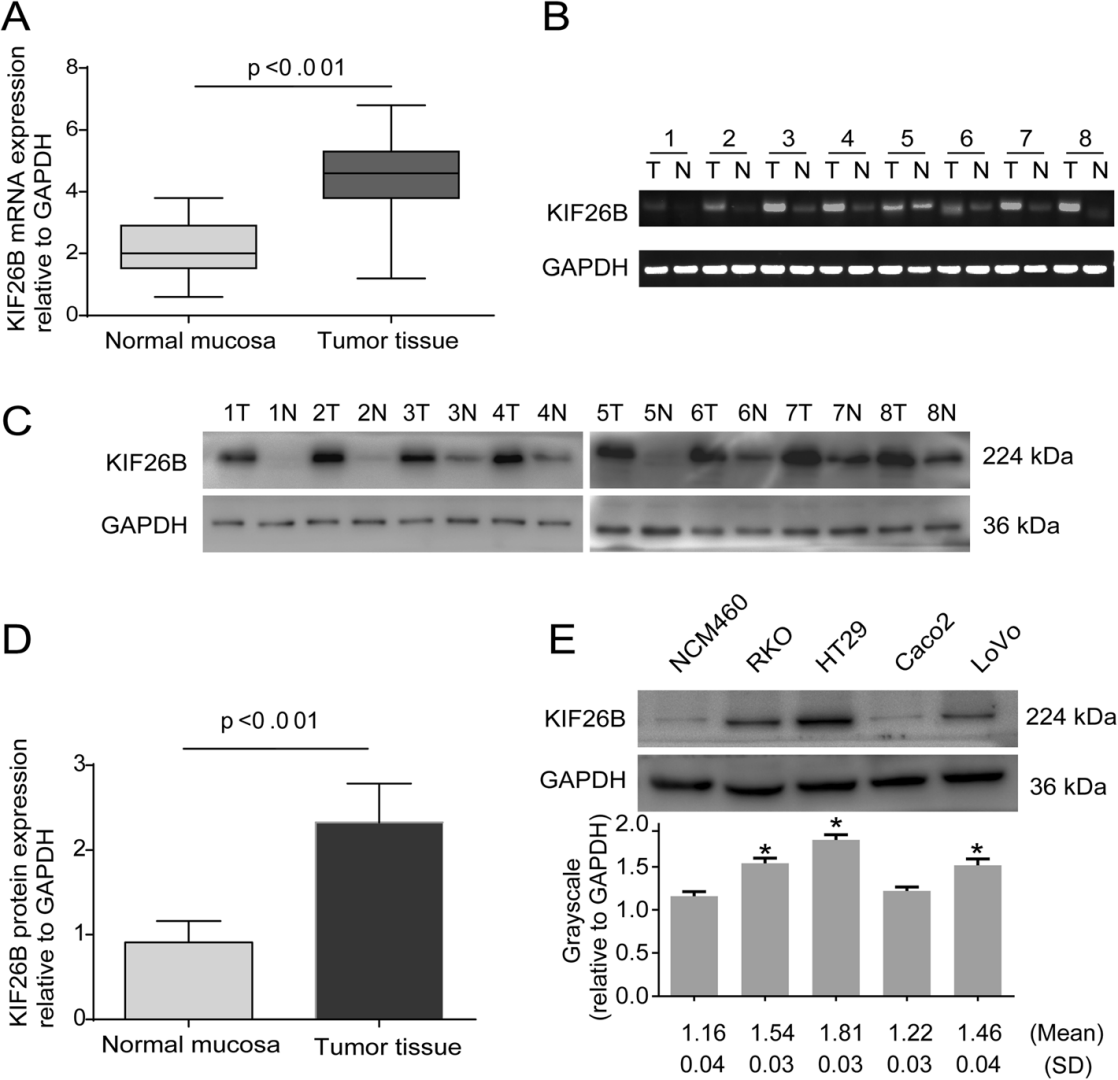
**Expression of KIF26B in human colorectal cancer tissues and cell lines.** KIF26B mRNA expression in 40 tumor tissues and paired adjacent normal mucosa **(A)**. RT PCR analysis of KIF26B mRNA expression in eight paired colorectal tumor tissues **(B)**. Western blot analysis of KIF26B expression in eight paired colorectal tumor tissues **(C)**. KIF26B protein is higher in tumor tissues than in paired adjacent normal mucosa **(D)**. KIF26B protein expression in five colorectal cell lines. Grayscale values were evaluated (n = 3, *P <0.05, compared with NCM460 cell line) **(E)**.

Forty paired specimens were analyzed for KIF26B mRNA and protein expression, 30 (75%) colorectal cancers showed a more than 2-fold increase in KIF26B mRNA levels compared with adjacent normal mucosa. The mean KIF26B mRNA expression was significantly higher in the 40 tumor tissue specimens compared with that in the paired adjacent normal mucosa specimens (4.35 ± 1.33 vs. 2.06 ± 0.86, respectively; *P* < 0.01, Student’s *t*-test) (Figure 1A–B). Western blot analysis also showed that KIF26B protein expression was significantly higher in the 40 tumor tissue specimens compared with that in the paired adjacent normal mucosa specimens (2.33 ± 0.07 vs. 0.91 ± 0.04, respectively; *P* < 0.01, Student’s *t*-test) (Figure 1C–D). KIF26B protein expression in the RKO, HT29 and LoVo cell lines was higher than that in the Caco2 and NCM460 cell lines (Figure 1E). These data indicated that KIF26B expression is commonly elevated in human CRC.

### Correlation between KIF26B overexpression and colorectal cancer clinicopathologic factors

**Figure 2:**
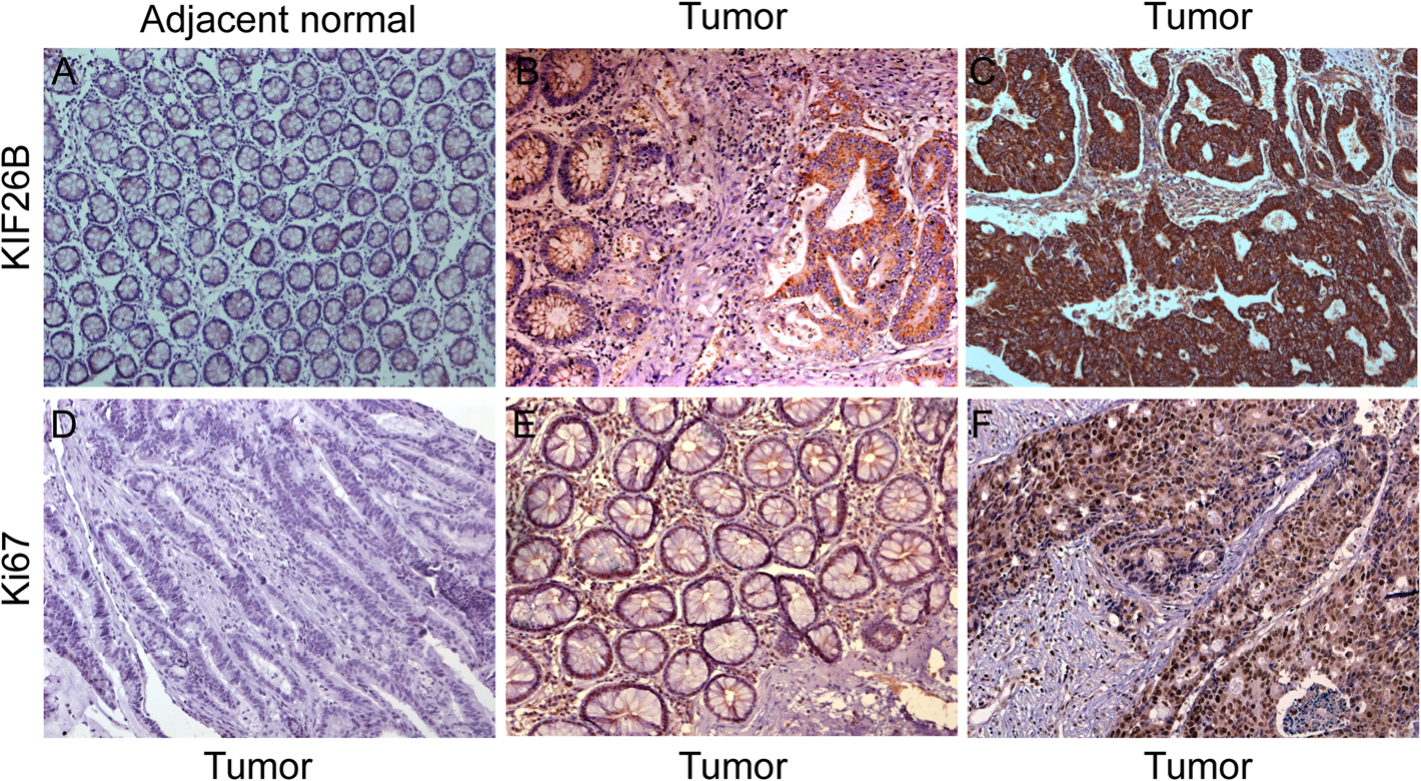
**Immunohistochemical staining for KIF26B and Ki67 expression in CRC cancer.** Adjacent normal mucosa showing very weak KIF26B staining (**A**, 200×), adjacent normal and tumor tissues showing weak and moderate staining respectively (**B**, 200×). Strong staining of KIF26B (**C**, 200×) in a moderately differentiated colon tumor. Negative control of Ki67 staining (**D**, 200×) in tumor tissues, Ki67-low staining (**E**, 200×) and Ki67-high staining (**F**, 200×) in tumor tissues.

KIF26B and Ki67 expression was determined by immunohistochemical analysis of a TMA containing 88 CRC specimens and paired adjacent normal mucosa. KIF26B expression was restricted to the cytoplasm with negligible nuclear staining, while Ki67 expression was restricted to the nucleus (Figure 2). Tumors showed variable (weak, moderate, and strong) KIF26B expression. High levels of KIF26B expression were detected in 53 of 88 CRC specimens. Associations of KIF26B and Ki67 expression with clinicopathological factors are summarized in Table 1. Upregulated KIF26B expression was significantly correlated with tumor size (*P* = 0.020), AJCC stage (*P* = 0.018), T stage (*P* = 0.026), N stage (*P* = 0.013), and histological differentiation (*P* = 0.047). High expression of Ki67 was significantly associated with AJCC stage (*P* = 0.015), T stage (*P* = 0.014), and N stage (*P* = 0.014). Furthermore, in most cases, tumors with high KIF26B expression showed high Ki67 expression. A positive correlation between KIF26B and Ki67 on the basis of proliferative activity was identified by using the Spearman’s correlation coefficient test (*P* < 0.01) (Table 2).

**Table 2:** **The association between KIF26B and Ki67 expression**

**Tissue sample**	**Ki67 expression**		***P*** **value**	**r**
	**Low**	**High**		
KIF26B Low	24	11		
KIF26B High	19	34	0.002	0.32

### Survival analysis and prognostic significance of KIF26B or Ki67 expression

**Figure 3:**
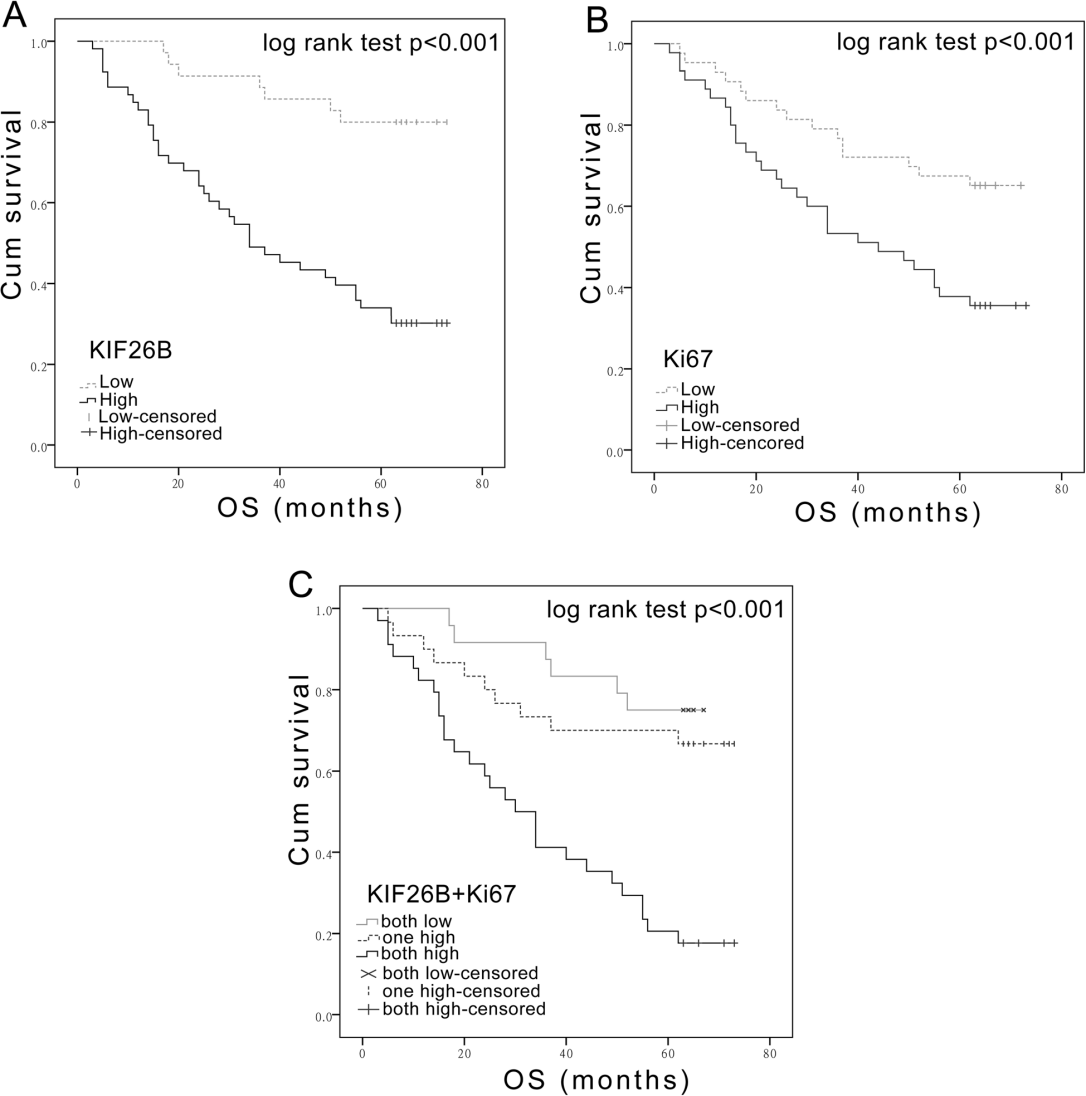
**Kaplan-Meier plots with log rank test of overall survival (OS).** Overall survival of 88 patients in relation to KIF26B expression levels **(A)** or Ki67 expression levels **(B)** determined by immunohistochemical staining of tissue microarrays. OS is significantly lower in patients with tumors expressing high levels of both KIF26B and Ki67 than that in patients with tumors expressing low levels of both KIF26B and Ki67 **(C)**.

Kaplan-Meier analysis with a log rank test for OS was performed to assess the possible association between tumor expression of KIF26B or Ki67 and patient survival (Figure 3). Patients with tumors expressing high levels of KIF26B had a poorer OS (*P* < 0.001) than patients with KIF26B-low tumors (Figure 3A), and patients with Ki67-high tumors had a lower OS (*P* < 0.001) than patients with a low Ki67 expression (Figure 3B). Furthermore, with regard to the concomitant expression of KIF26B and Ki67 proteins, we divided the samples into three groups as follows: Group 1, tumors exhibiting both high expression of KIF26B and Ki67; Group 2, tumors with high expression of only one protein; Group 3, tumors with low expression of both proteins. Notably, a better OS was observed in Group 3 compared to that of Group 2, while the poorest OS was observed in Group 1 (*P* < 0.001) (Figure 3C).

Using a univariate analysis in the Cox proportional hazards model, a decreased OS was associated with the following characteristics: tumor location, tumor depth, AJCC stage, LNM stage, distant metastasis, vascular invasion and the expression of KIF26B and Ki67 (Table 3). Multivariate analysis revealed that the expression of KIF26B is an independent prognostic factor for OS (HR 5.621; 95% CI 2.302–13.730; *P* < 0.001).

**Table 3:** **Univariate and multivariate analysis of overall survival**

	**Univariate analysis**		**Multivariate analysis**	
	**HR (95% CI)**	***P*** **value**	**HR (95% CI)**	***P*** **Value**
Age				
<65	1			
≥65	1.350(0.706-2.581)	0.364		
Gender				
Male	1			
Female	0.910(0.503-1.648)	0.756		
Location				
Right	1		1	
Others	0.470(0.259-0.852)	0.013*	0.501(0.272-0.922)	0.026*
Tumor size (cm)				
< 5	1			
≥ 5	1.801(0.993-3.267)	0.053		
AJCC stage				
I + II	1		1	
III + IV	3.106(1.701-5.671)	<0.001*	82.935(13.868-495.998)	<0.001*
T stage				
T1 + T2	1			
T3 + T4	1.662(0.514-5.370)	0.396		
N stage				
N0	1		1	
N1 + N2	3.106(1.701-5.671)	<0.001*	2.558(1.355-4.828)	0.004*
M stage				
M0	1		1	
M1	10.801(2.448-47.658)	0.002*	77.710(13.384-451.213)	<0.001*
Differentiation				
Well + Moderate	1			
Poor	2.026(0.627-6.548)	0.238		
Vascular invasion				
No	1		1	
Yes	6.188(2.093-18.295)	<0.001*	1.111(0.246-5.019)	0.892
KIF26B				
Low	1		1	
High	5.311(2.360-11.952)	<0.001*	5.621(2.302-13.730)	<0.001*
Ki67				
Low	1		1	
High	2.343(1.254-4.377)	0.008*	1.433(0.718-2.860)	0.307

*indicates *P*<0.05.

### Knockdown of KIF26B expression inhibits colorectal cancer cell proliferation

**Figure 4:**
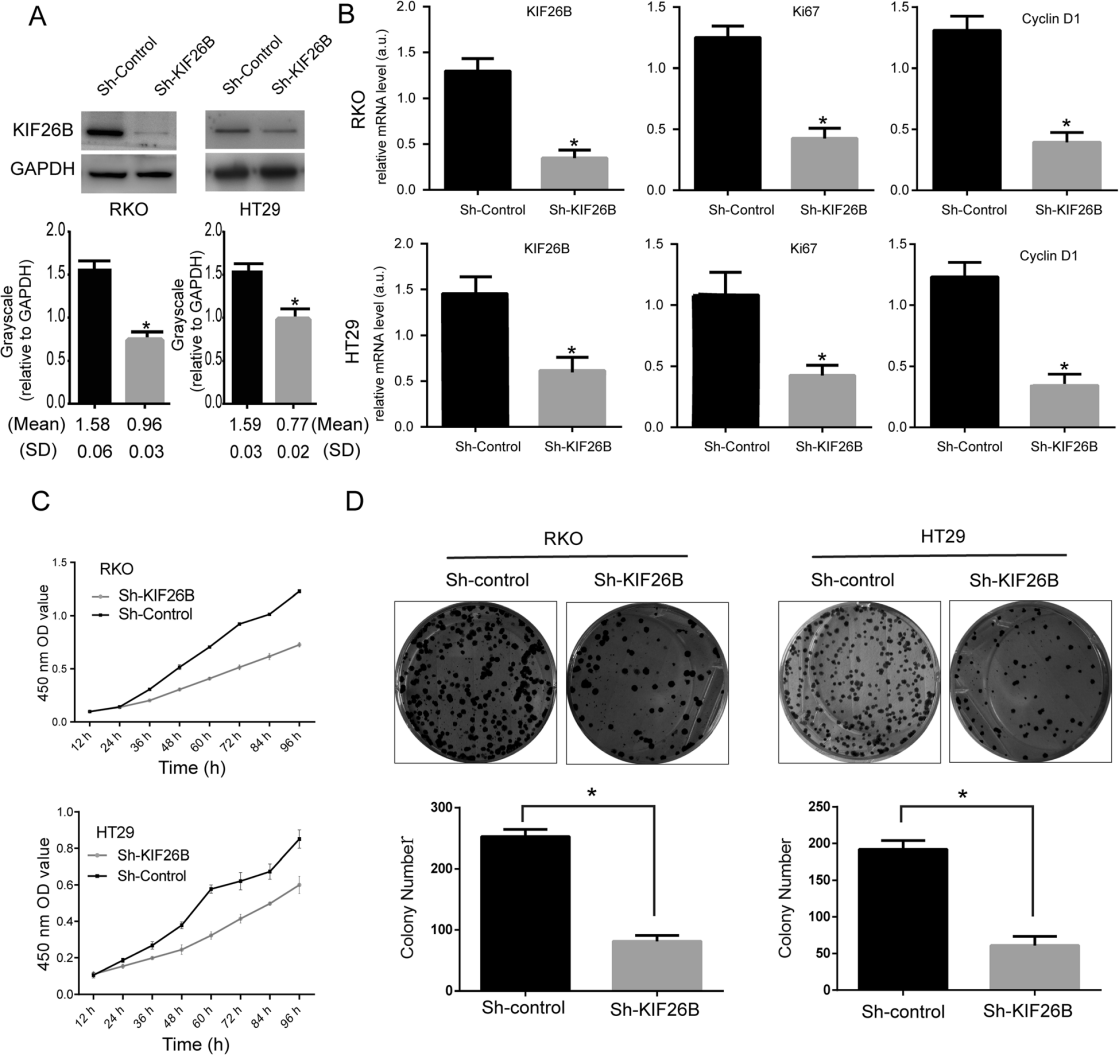
**KIF26B knockdown inhibits cancer cell proliferation.** Western blot analysis of KIF26B protein expression in stable knockdown RKO and HT29 cell lines. Grayscale values were evaluated (n = 3, *P <0.05) **(A)**. Expression of proliferation-related genes was inhibited in KIF26B knockdown cells according to real-time PCR analysis (n = 3; *P < 0.05) **(B)**. Effects of KIF26B knockdown on cell growth was evaluated by Cell Counting Kit-8 assays **(C)** and plate colony formation assays **(D)** (n = 3; *P < 0.05).

To further investigate the potential effects of KIF26B on CRC cell proliferation, we used KIF26B-shRNA to knockdown expression in transfected RKO and HT29 cell lines. Control-shRNA plasmids were used as controls and stable transfected cell lines were obtained by puromycin selection. The efficacy of KIF26B knockdown was confirmed by Western blot and real-time PCR analyses (Figure 4A–B). To evaluate the effects of KIF26B knockdown on CRC cell proliferation, the expression of proliferation-related genes (Ki67; cyclinD1) was detected by real-time PCR analysis (Figure 4B). The expression of both Ki67 and cyclinD1 mRNA was downregulated in KIF26B-shRNA cells. Next, we performed CCK-8 and plate colony formation assays to assess the role of KIF26B in CRC cell growth (Figure 4C–D). As shown in Figure 4C, KIF26B knockdown was associated with significantly decreased cell proliferation compared with that of control-shRNA cells. Furthermore, KIF26B knockdown in CRC cells consistently reduced the colony formation ability compared with control-shRNA cells (*P* < 0.01). These results indicated that KIF26B plays a role in CRC cell proliferation.

## Discussion

Numerous studies have shown that the abnormal expression and function of kinesins plays a key role in the development and progression of many human cancers [16,17]. KIF26B comprises 2,108 amino acids, with a predicted molecular weight of 223.8 kDa. In mice, KIF26B plays a role in embryogenesis, specifically in the development of limbs, faces, and somites [26]. KIF26B also plays an important role in kidney development and is involved in the development and progression of some types of tumors, including breast cancer, esophageal adenocarcinoma, and colorectal adenomatous polyposis [19,22-24]. However, studies on KIF26B expression during tumorigenesis and progression of CRC are rare.

The present study showed for the first time that both KIF26B mRNA and protein expression are upregulated in primary CRC tissues. In the immunohistochemical analysis, high KIF26B expression in colorectal cancer tissues was significantly associated with other malignant tumor characteristics, such as larger tumor size, AJCC stage, tumor depth, differentiation histology, and lymph node metastasis, implicating KIF26B as a biomarker of malignant tumors. Ki67 is one of the most commonly used markers to evaluate the proliferation of tumor cells and has prognostic value in some types of cancer [27-30]. In our study, we found that high KIF26B expression correlated positively with Ki67 expression, suggesting that KIF26B is involved in tumor cell proliferation.

Given that kinesins are involved in mitosis, KIFs have attracted significant attention in the search for novel, alternative mitotic drug targets of cancer [31-33]. Several KIF11-inihibitors have entered Phase I or Phase II clinical trials either in combination with other drugs or as monotherapies [16]. Vaccination with a KIF20A-derived peptide in combination with gemcitabine is a feasible and promising approach to the treatment of advanced pancreatic cancer [34]. We hypothesized that knockdown of KIF26B will reduce cancer cell proliferation and is therefore, a potential therapeutic target. In this study, investigation of the effect of KIF26B on CRC cell proliferation in CCK-8 assays showed that KIF26B knockdown in RKO and HT29 cells inhibited cell proliferation activity. In addition, expression of KIF26B and proliferation-related genes (Ki67; cyclinD1) was shown to significantly downregulated in KIF26B knockdown cells using quantitative real-time PCR assays. Moreover, colony formation ability was significantly reduced in KIF26B knockdown cells. These data indicate that suppression of KIF26B inhibits cell proliferation and implicate KIF26B as a potential therapeutic target for CRC.

To date, the prognostic value of KIF26B expression in CRC has not been reported. Our Kaplan-Meier survival analysis demonstrated that high KIF26B expression is significantly related to poor prognosis after surgical resection in CRC patients (*P* < 0.001). In our study, patients with elevated KIF26B expression were significantly linked to poorer OS. Furthermore, Cox regression analysis suggested that KIF26B is an independent prognostic factor, indicating that KIF26B is a new predictor to the prognosis of patients with CRC.

It should be noted that there are some limitations of our study. First, KIF26B overexpressing cell lines were not investigated in our study. Despite repeated attempts, we were unable to generate a KIF26B overexpression plasmid because of the huge coding sequence (CDS, 6,315 bp, GeneBank, NM_018012). Second, the effects of KIF26B knockdown were not investigated in vivo in the present study. Third, further studies are required to confirm our hypothesis that KIF26B is a potential target for CRC therapy.

## Conclusion

Taken together, our data indicate that KIF26B plays an important role in CRC carcinogenesis, especially in tumor development, progression and proliferation. Our study demonstrates for the first time that KIF26B expression is elevated in CRC tissues at both the mRNA and protein levels. Furthermore, KIF26B knockdown inhibited cell proliferation in CRC cell lines. Moreover, our study provides clinical evidence that KIF26B is an independent prognostic indicator of outcome for CRC patients; thus, KIF26B is implicated as a potential therapeutic target in CRC. Further investigations are required to fully elucidate the role of KIF26B in the progression of CRC and the potential of KIF26B as a therapeutic target.

## References

[1] Jemal A, Bray F, Center MM, Ferlay J, Ward E, Forman D (2011). Global cancer statistics. CA Cancer J Clin.

[2] Siegel R, Naishadham D, Jemal A (2013). Cancer statistics, 2013. CA Cancer J Clin.

[3] DeSantis CE, Lin CC, Mariotto AB, Siegel RL, Stein KD, Kramer JL (2014). Cancer treatment and survivorship statistics, 2014. CA Cancer J Clin.

[4] Stracci F, Zorzi M, Grazzini G (2014). Colorectal cancer screening: tests, strategies, and perspectives. Front Public Health.

[5] Kuipers EJ, Rosch T, Bretthauer M (2013). Colorectal cancer screening–optimizing current strategies and new directions. Nat Rev Clin Oncol.

[6] Xu JM, Liu XJ, Ge FJ, Lin L, Wang Y, Sharma MR (2014). KRAS mutations in tumor tissue and plasma by different assays predict survival of patients with metastatic colorectal cancer. J Exp Clin Cancer Res.

[7] Anaya DA, Becker NS, Abraham NS (2011). Global graying, colorectal cancer and liver metastasis: new implications for surgical management. Crit Rev Oncol Hematol.

[8] Tonini G, Imperatori M, Vincenzi B, Frezza AM, Santini D (2013). Rechallenge therapy and treatment holiday: different strategies in management of metastatic colorectal cancer. J Exp Clin Cancer Res.

[9] Miki H, Setou M, Kaneshiro K, Hirokawa N (2001). All kinesin superfamily protein, KIF, genes in mouse and human. Proc Natl Acad Sci U S A.

[10] Lawrence CJ, Dawe RK, Christie KR, Cleveland DW, Dawson SC, Endow SA (2004). A standardized kinesin nomenclature. J Cell Biol.

[11] Hirokawa N, Noda Y, Tanaka Y, Niwa S (2009). Kinesin superfamily motor proteins and intracellular transport. Nat Rev Mol Cell Biol.

[12] Dagenbach EM, Endow SA (2004). A new kinesin tree. J Cell Sci.

[13] Sablin EP (2000). Kinesins and microtubules: their structures and motor mechanisms. Curr Opin Cell Biol.

[14] Hirokawa N (1998). Kinesin and dynein superfamily proteins and the mechanism of organelle transport. Science.

[15] Miki H, Okada Y, Hirokawa N (2005). Analysis of the kinesin superfamily: insights into structure and function. Trends Cell Biol.

[16] Rath O, Kozielski F (2012). Kinesins and cancer. Nat Rev Cancer.

[17] Yu Y, Feng YM (2010). The role of kinesin family proteins in tumorigenesis and progression: potential biomarkers and molecular targets for cancer therapy. Cancer.

[18] Zhou R, Niwa S, Homma N, Takei Y, Hirokawa N (2009). KIF26A is an unconventional kinesin and regulates GDNF-Ret signaling in enteric neuronal development. Cell.

[19] Uchiyama Y, Sakaguchi M, Terabayashi T, Inenaga T, Inoue S, Kobayashi C (2010). Kif26b, a kinesin family gene, regulates adhesion of the embryonic kidney mesenchyme. Proc Natl Acad Sci U S A.

[20] Nishinakamura R, Uchiyama Y, Sakaguchi M, Fujimura S (2011). Nephron progenitors in the metanephric mesenchyme. Pediatr Nephrol.

[21] Heinrich J, Proepper C, Schmidt T, Linta L, Liebau S, Boeckers TM (2012). The postsynaptic density protein Abelson interactor protein 1 interacts with the motor protein Kinesin family member 26B in hippocampal neurons. Neuroscience.

[22] Wang Q, Zhao ZB, Wang G, Hui Z, Wang MH, Pan JF (2013). High expression of KIF26B in breast cancer associates with poor prognosis. PLoS One.

[23] Gu J, Ajani JA, Hawk ET, Ye Y, Lee JH, Bhutani MS (2010). Genome-wide catalogue of chromosomal aberrations in barrett’s esophagus and esophageal adenocarcinoma: a high-density single nucleotide polymorphism array analysis. Cancer Prev Res (Phila).

[24] Horpaopan S, Spier I, Zink AM, Altmuller J, Holzapfel S, Laner A (2015). Genome-wide CNV analysis in 221 unrelated patients and targeted high-throughput sequencing reveal novel causative candidate genes for colorectal adenomatous polyposis. Int J Cancer.

[25] O’Connell JB, Maggard MA, Ko CY (2004). Colon cancer survival rates with the new American Joint Committee on Cancer sixth edition staging. J Natl Cancer Inst.

[26] Marikawa Y, Fujita TC, Alarcon VB (2004). An enhancer-trap LacZ transgene reveals a distinct expression pattern of Kinesin family 26B in mouse embryos. Dev Genes Evol.

[27] Yerushalmi R, Woods R, Ravdin PM, Hayes MM, Gelmon KA (2010). Ki67 in breast cancer: prognostic and predictive potential. Lancet Oncol.

[28] Allegra CJ, Paik S, Colangelo LH, Parr AL, Kirsch I, Kim G (2003). Prognostic value of thymidylate synthase, Ki-67, and p53 in patients with Dukes’ B and C colon cancer: a National Cancer Institute-National Surgical Adjuvant Breast and Bowel Project collaborative study. J Clin Oncol.

[29] Brown DC, Gatter KC (2002). Ki67 protein: the immaculate deception?. Histopathology.

[30] Amini A, Masoumi-Moghaddam S, Ehteda A, Morris DL (2014). Bromelain and N-acetylcysteine inhibit proliferation and survival of gastrointestinal cancer cells in vitro: significance of combination therapy. J Exp Clin Cancer Res.

[31] Huszar D, Theoclitou ME, Skolnik J, Herbst R (2009). Kinesin motor proteins as targets for cancer therapy. Cancer Metastasis Rev.

[32] Liu X, Gong H, Huang K (2013). Oncogenic role of kinesin proteins and targeting kinesin therapy. Cancer Sci.

[33] Jiang C, You Q (2013). Kinesin spindle protein inhibitors in cancer: a patent review (2008 - present). Expert Opin Ther Pat.

[34] Suzuki N, Hazama S, Ueno T, Matsui H, Shindo Y, Iida M (2014). A phase I clinical trial of vaccination with KIF20A-derived peptide in combination with gemcitabine for patients with advanced pancreatic cancer. J Immunother.

